# Supplementary material for: Crystal structures of U6 snRNA-specific terminal uridylyltransferase
Source: Nat Commun. 2017 Jun 7;8:15788. doi: 10.1038/ncomms15788 (PMC5467268; doi:10.1038/ncomms15788)
Supplement: Supplementary Information — Supplementary figures, supplementary tables and supplementary references. [file ncomms15788-s1.pdf]

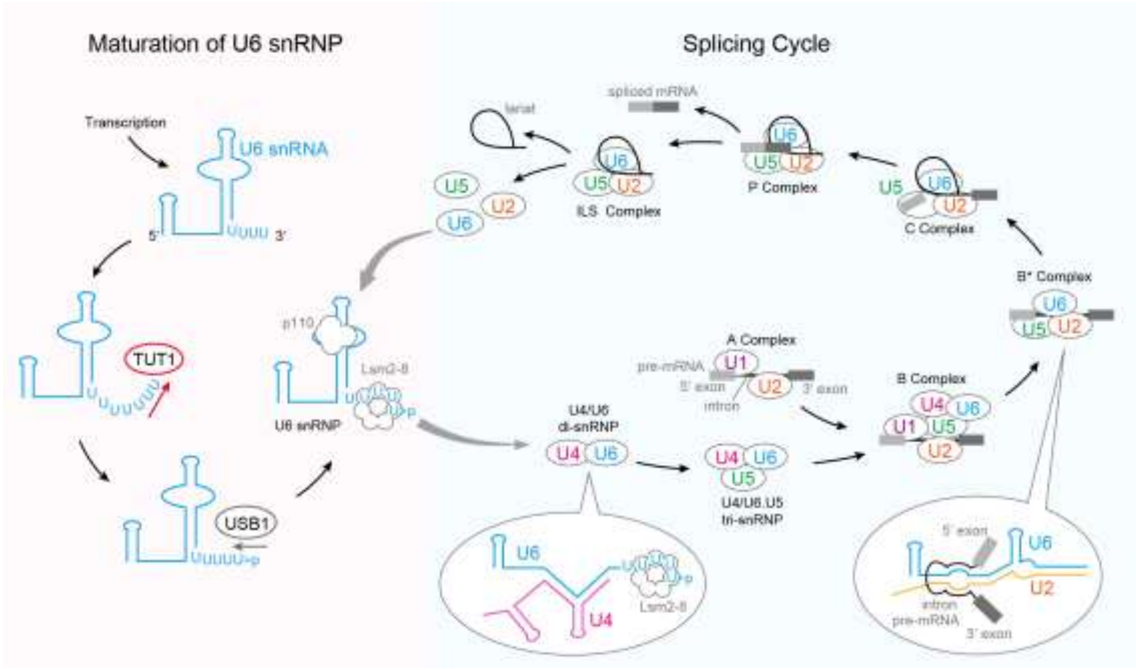

Supplementary Fig. 1: Schematic diagram of the maturation and recycling processes of U6 snRNA.

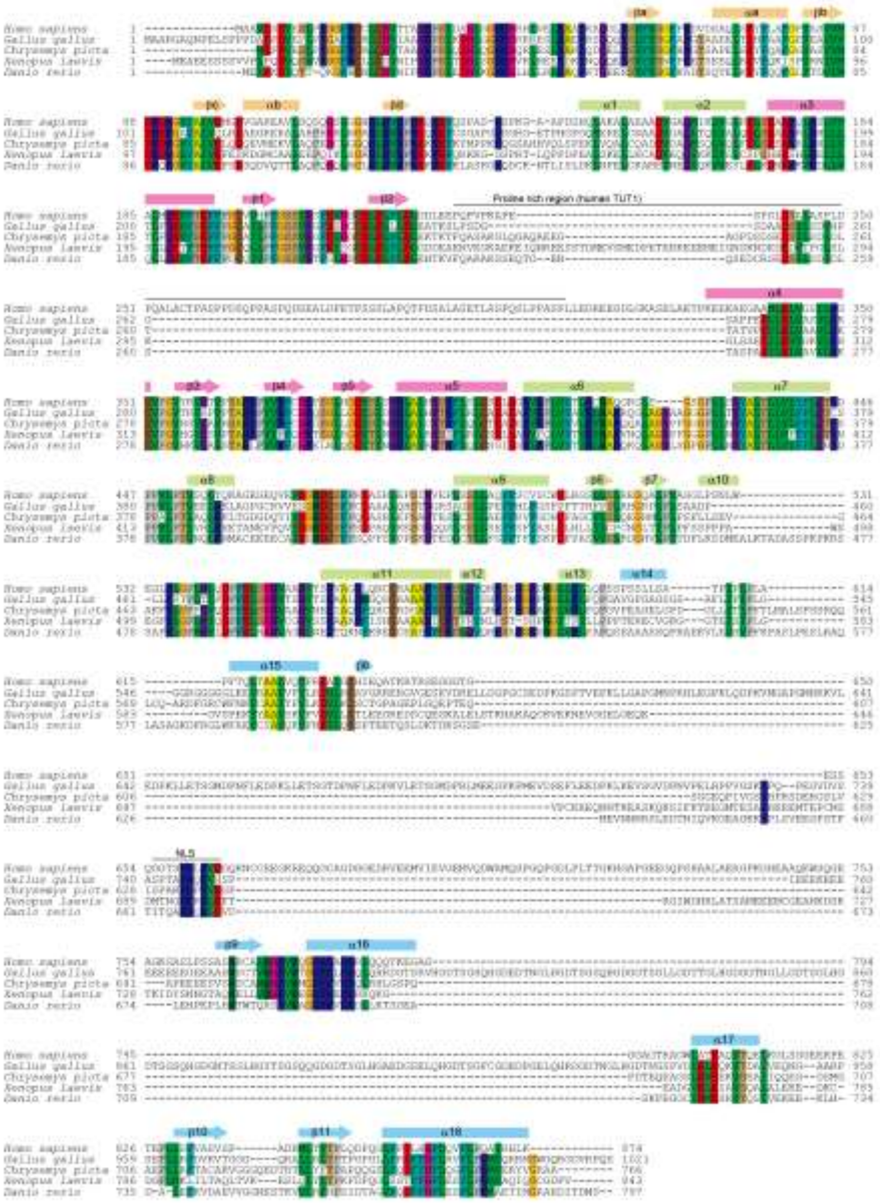

Supplementary Fig. 2: Sequence alignment of the vertebrate TUT1 proteins. The human TUT1 sequence (*Homo sapiens*; Q9H6E5.2) was aligned with the following sequences: *Gallus gallus* (XP_015128520.1); *Chrysemys picta* (XP_008172327.1); *Xenopus laevis* (XP_002941502.2) and *Danio rerio* (NP_001025359.1). Secondary structures (α-helices and β-sheets) of human TUT1 domains, revealed by the crystal structures determined in the present study, are depicted above the sequences: RRM (orange), Palm domain (magenta), Fingers domain (green) and KA-1 domain (cyan). PRR and NSL are underlined.

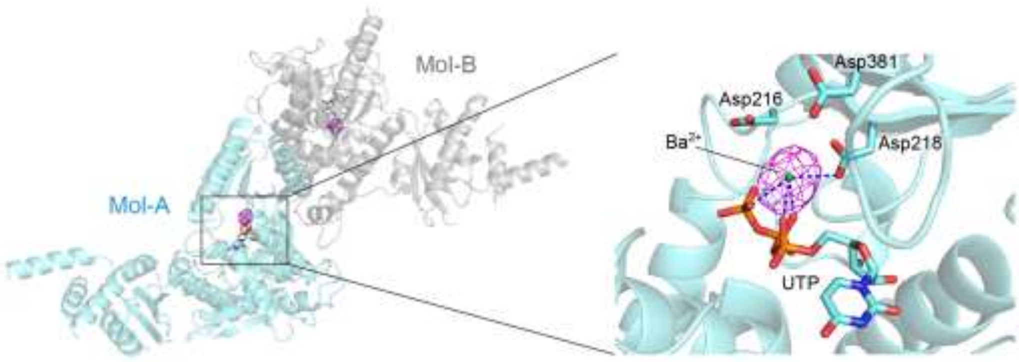

Supplementary Fig. 3: Identification of the barium ion (Ba^{2+}) by BaUTP soaking into the TUT1 form-I crystal. The anomalous difference Fourier map contoured at 5.0 σ (colored magenta), overlaid on the active sites of Mol-A and Mol-B in the TUT1_ΔN structures (left). A detailed view of the active site in Mol-A (right). Ba^{2+} is colored green.

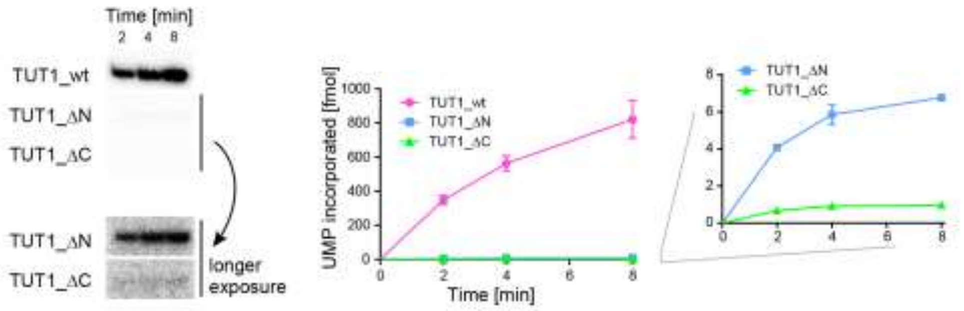

Supplementary Fig. 4: UMP incorporations by TUT1 variants used for crystallization. Time courses of UMP incorporations into U6 snRNA-u4 under the standard conditions. Wild-type TUT1, TUT1_ΔN (141-874, Δ235-304, Δ651-750, C372A/C399A/C415A/C501A/C504S/C574A), and TUT1_ΔC (54-599, Δ235-304, C372A/C399A/C415A/C501A/C504S/C574A) were incubated with U6 snRNA-4u and α-^{32}P UTP, as in Fig. 2d. The reaction products were separated by 10%(w/v) polyacrylamide gel electrophoresis under denaturing conditions.

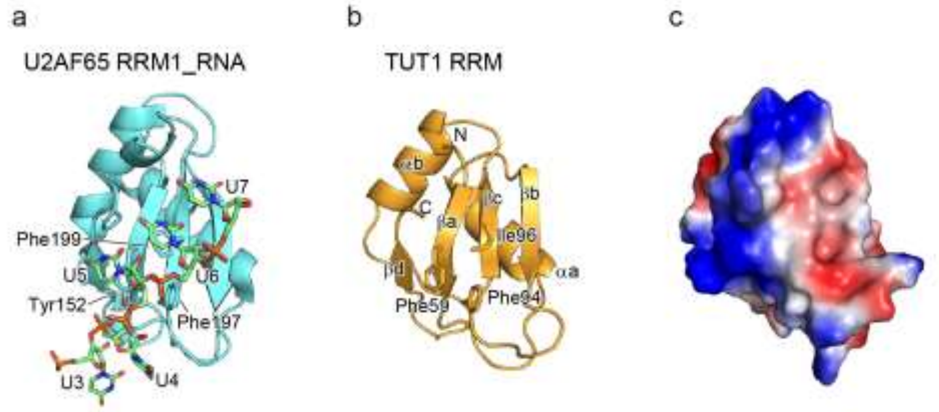

Supplementary Fig. 5: Structure of N-terminal RRM of TUT1. (a) Structure of the U2AF65 RRM1 complexed with RNA (PDB ID: 2G4B) [1]. In RRM family proteins, three well conserved aromatic residues (Tyr152, Phe197, and Phe199 in U2AF65 RRM1) are involved in single-stranded RNA binding. **(b,c)** Cartoon representation **(b)** and electrostatic surface **(c)** of the TUT1 RRM. The overall domain fold of the TUT1 RRM is similar to that of U2AF65, and aromatic (or hydrophobic) residues are conserved in the TUT1 RRM (Phe59, Phe94 and Ile96). The regions around αb and βd have positively charged surfaces.

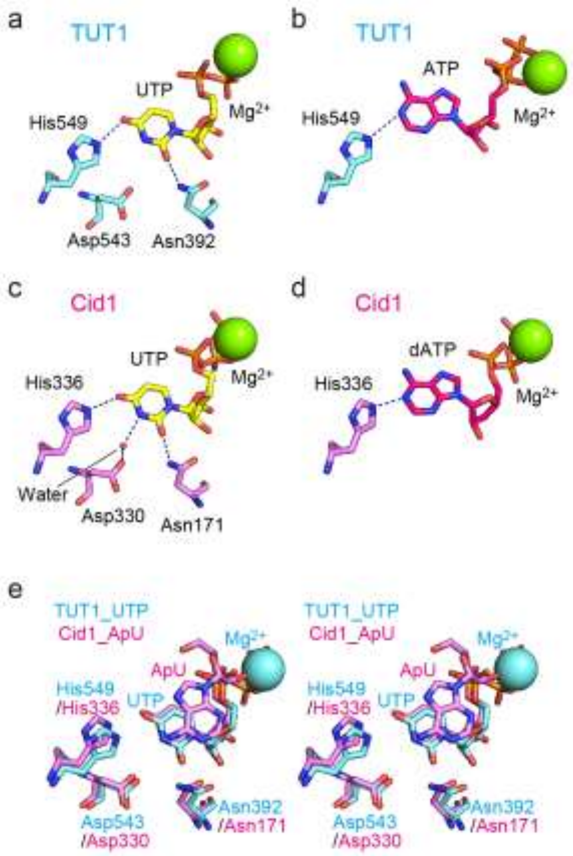

Supplementary Fig. 6: Nucleotide recognition by human TUT1 and yeast Cid1. (a) UTP recognition by human TUT1. **(b)** ATP recognition by human TUT1. **(c)** UTP recognition by yeast Cid1. The N_3 atom of UTP forms a hydrogen-bond with a water-molecule, which also forms a hydrogen-bond with the side chain of Asp330. **(d)** dATP recognition by yeast Cid1[2-4]. Although the water molecule was not observed in our TUT1 structure complexed with UTP (a), due to the low resolution of the dataset, the conserved Asp543 would be involved in the water-mediated interaction with the N_3 atom of UTP, as in yeast Cid1. **(e)** Superimposition of the structures of TUT1 complexed with UTP (Fig. 2b, c) and yeast Cid1 complexed with ApU[5].

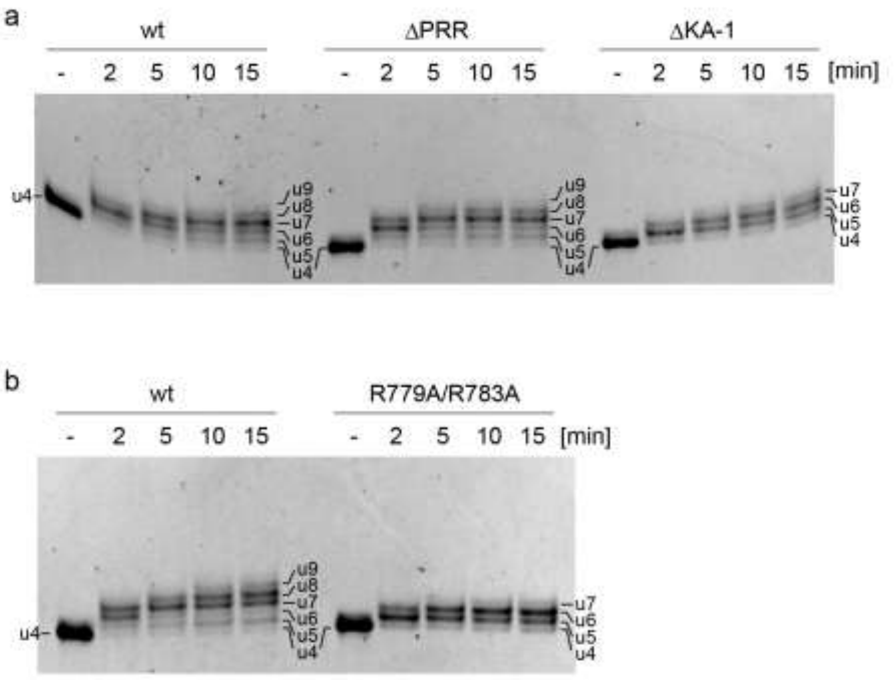

Supplementary Fig. 7: Oligo-uridylylation of U6 snRNA-U4 by TUT1 and its variants. The reaction solutions contained 50 nM U6 snRNA-u4 and 50 nM wild-type TUT1 (or its variants). The reaction was stopped at the indicated time, and the products were separated on a sequencing gel under denaturing conditions. The gel was stained with ethidium bromide. **(a)** The reaction products of wild-type TUT1, ΔPRR, and ΔKA-1. **(b)** The reaction products of wild-type TUT1 and the R779A/R783A mutant.

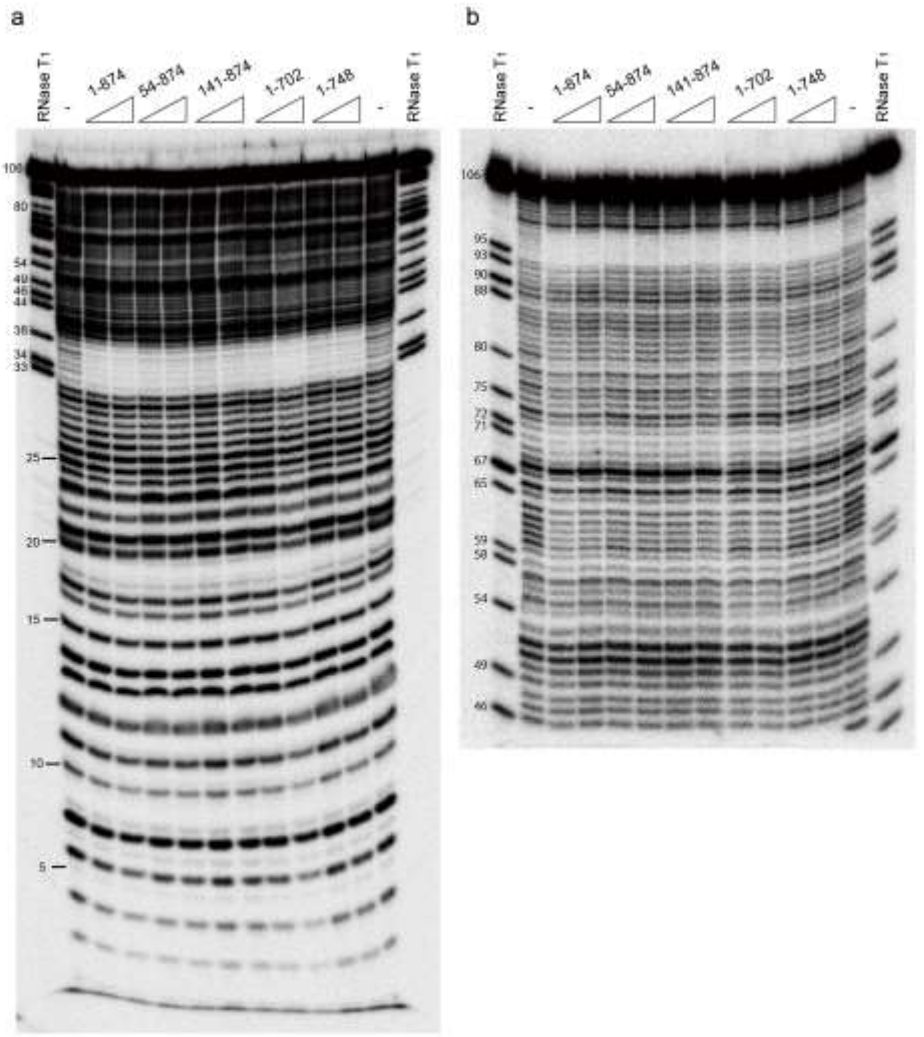

Supplementary Fig. 8: Interaction of human TUT1 with U6 snRNA. Tb(III)-mediated cleavage patterns of U6 snRNA in the absence and presence of full-length TUT1 (amino acid residues 1-874) and truncated TUT1 proteins. The numbers above the gels indicate the amino acid residues of the TUT proteins used for the footprinting. ^{32}P-labeled U6 snRNA-u4 was incubated with Tb(III) in the absence or presence of 0.4 µM and 0.8 µM recombinant full-length TUT1 and its variants. The positions of the U6 snRNA nucleotides were determined by partial digestion of the RNA substrate with RNase T_1. Cleavage patterns were analyzed on 16%(w/v) **(a)** and 8%(w/v) **(b)** sequencing gels. Protected and de-protected regions were quantified with a BAS-5000 imager (Fuji Film, Japan).

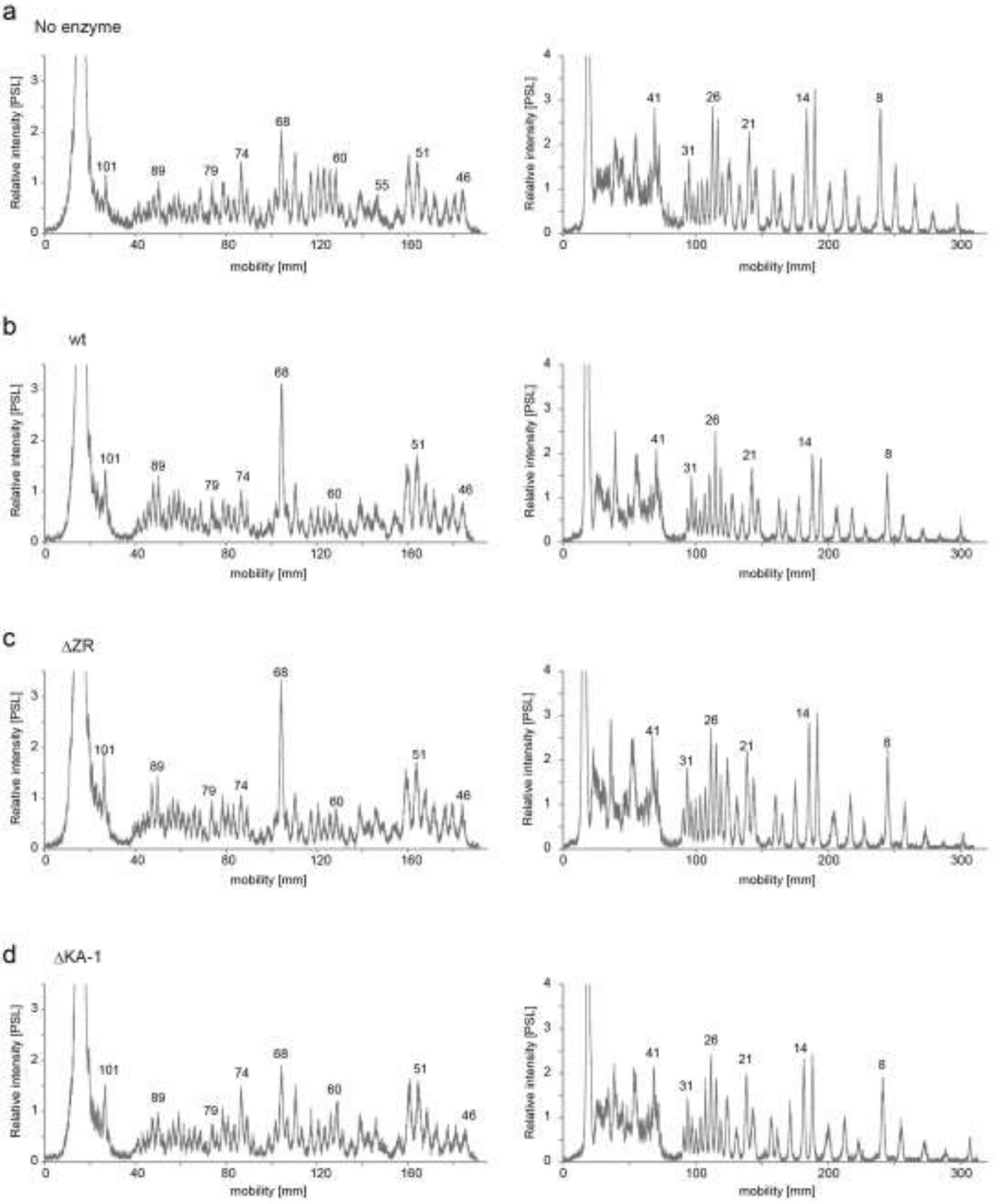

Supplementary Fig. 9: Band intensity profiles of foot-printing assay. The autoradiographs in Supplementary Fig. 8 were quantified with the Image Gauge software (Fuji Film, Japan). The intensity profiles from the lanes of (**a**) No enzyme, (**b**) wild-type, (**c**) ΔZR, and (**d**) ΔKA-1 are shown. The numbers in the graphs indicate the nucleotide positions of U6 snRNA.

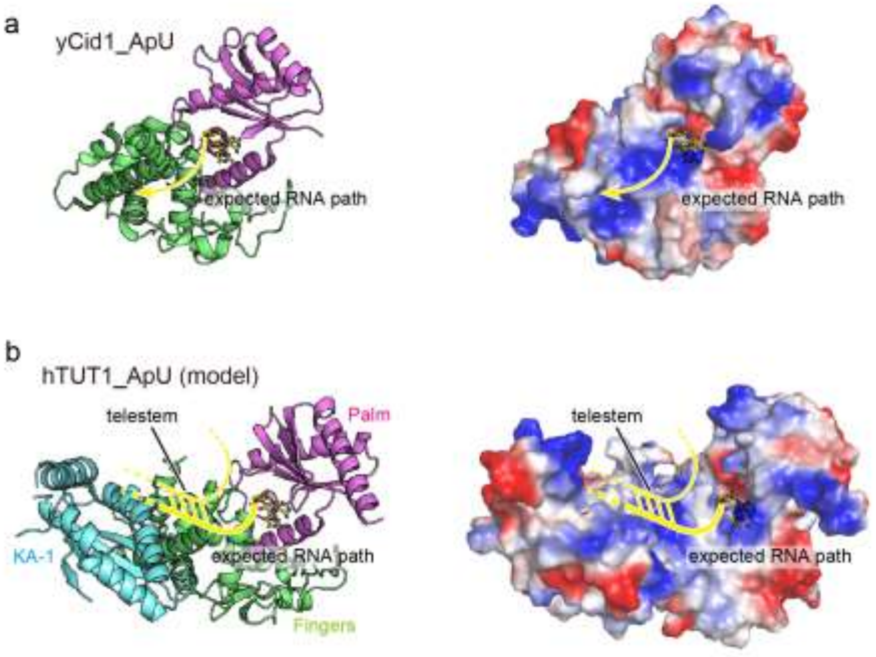

Supplementary Fig. 10: A model of the 3'-end binding site on TUT1.

(**a**) Cartoon representation (left) and electrostatic surface (right) of yeast Cid1 complexed with ApU. The expected RNA path is depicted by the yellow arrow[3]. (**b**) Cartoon representation (left) and electrostatic surface (right) of the model of the TUT1-ApU complex. The telestem of U6 snRNA would be crumpled in the cleft between the palm (magenta) and fingers (green) domains, and the unfolded 3'-oligo(U) region enters the catalytic site, as modeled in Fig. 5.

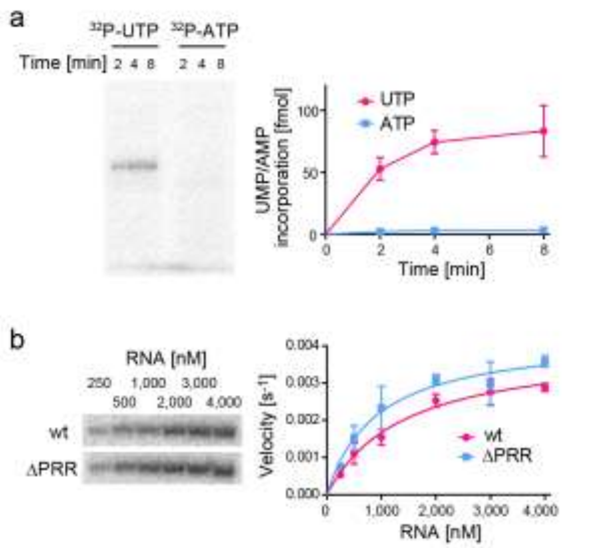

Protein	RNA	Km [nM]	kcat [s⁻¹]	Km / kcat (Relative)
wt	U6 snRNA-u4	55 ± 22	0.061 ± 0.007	100
ΔPRR	U6 snRNA-u4	55 ± 17	0.058 ± 0.005	95
wt	3'-UTR-HO1	1410 ± 280	0.0040 ± 0.0003	0.26
ΔPRR	3'-UTR-HO1	910 ± 240	0.0043 ± 0.0004	0.43

Supplementary Fig. 11: Nucleotide incorporation onto the 3'-end of 3'-UTR-HO1 RNA. (a) Time-courses of UTP or ATP incorporation onto the 3'-end of the 3'-UTR-HO1 transcript by wild-type TUT1, under standard conditions (1 μM RNA). **(b)** Steady-state kinetics of UMP incorporation by wild-type TUT1 and the ΔPRR variant into the 3'-UTR-HO1 transcript, with various RNA concentrations (250-4,000 nM). Bars in the graphs indicate SD of two independent experiments. Table (below) of kinetic parameters of UMP incorporation into U6 snRNA-u4 and 3'-UTR-HO1, by wild-type TUT1 and ΔPRR. The U6 snRNA is a much better substrate than the 3'-UTR-HO1 RNA *in vitro*.

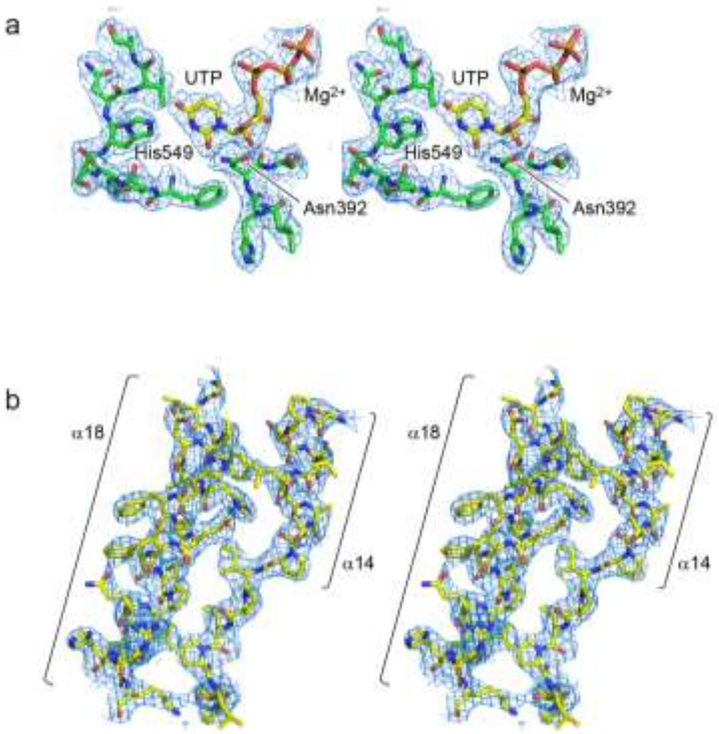

Supplementary Fig. 12: Representative images of the electron density.

Stereo views of the 2m*F*o-D*F*c maps of TUT1_ΔN bound with MgUTP, contoured at 1.0 σ. The electron densities of **(a)** the residues around MgUTP and **(b)** α14 and α 18 helices in the KA-1 domain are shown in blue.

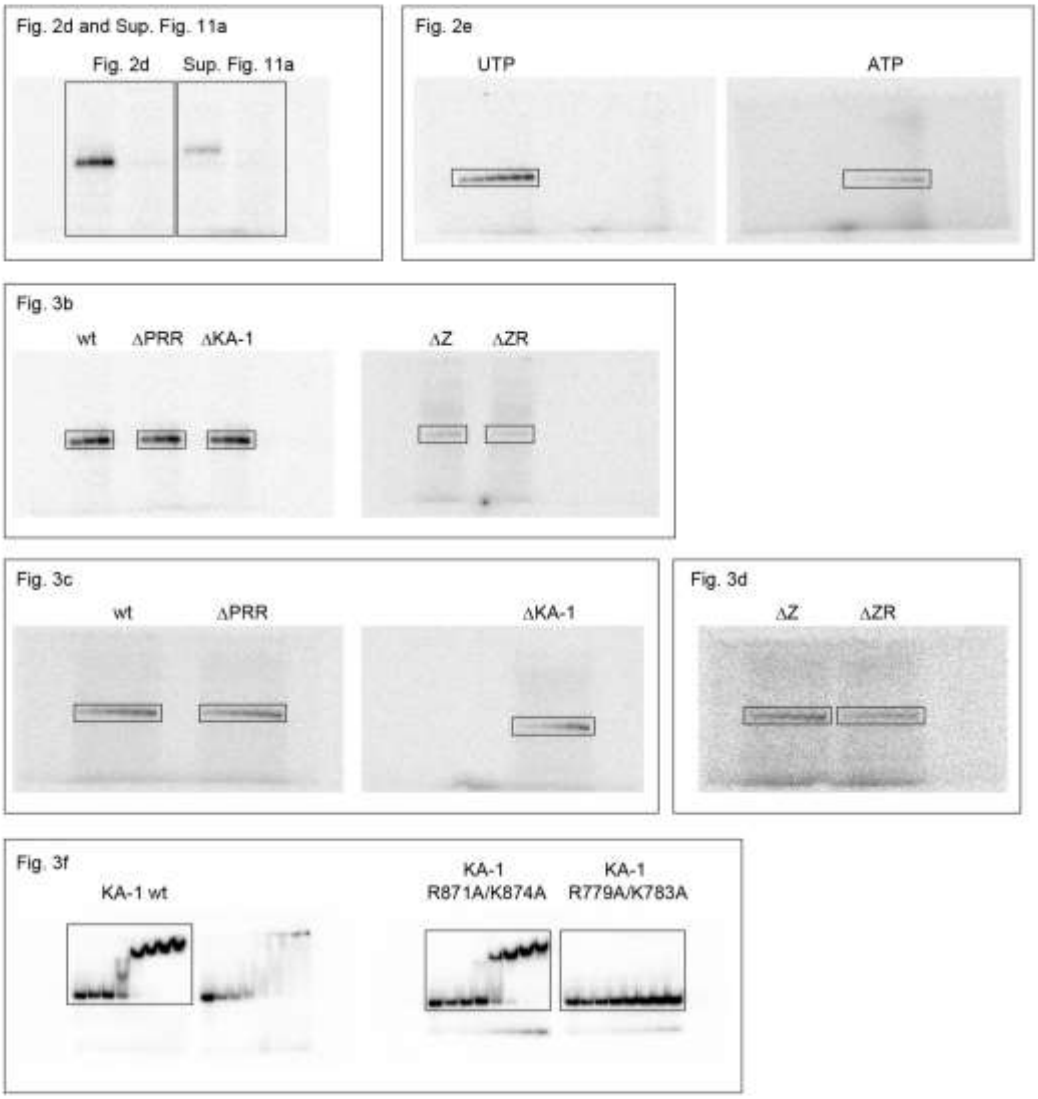

Supplementary Fig. 13: Uncropped images of Figs. 2 and 3, and Supplementary Fig. 11a.

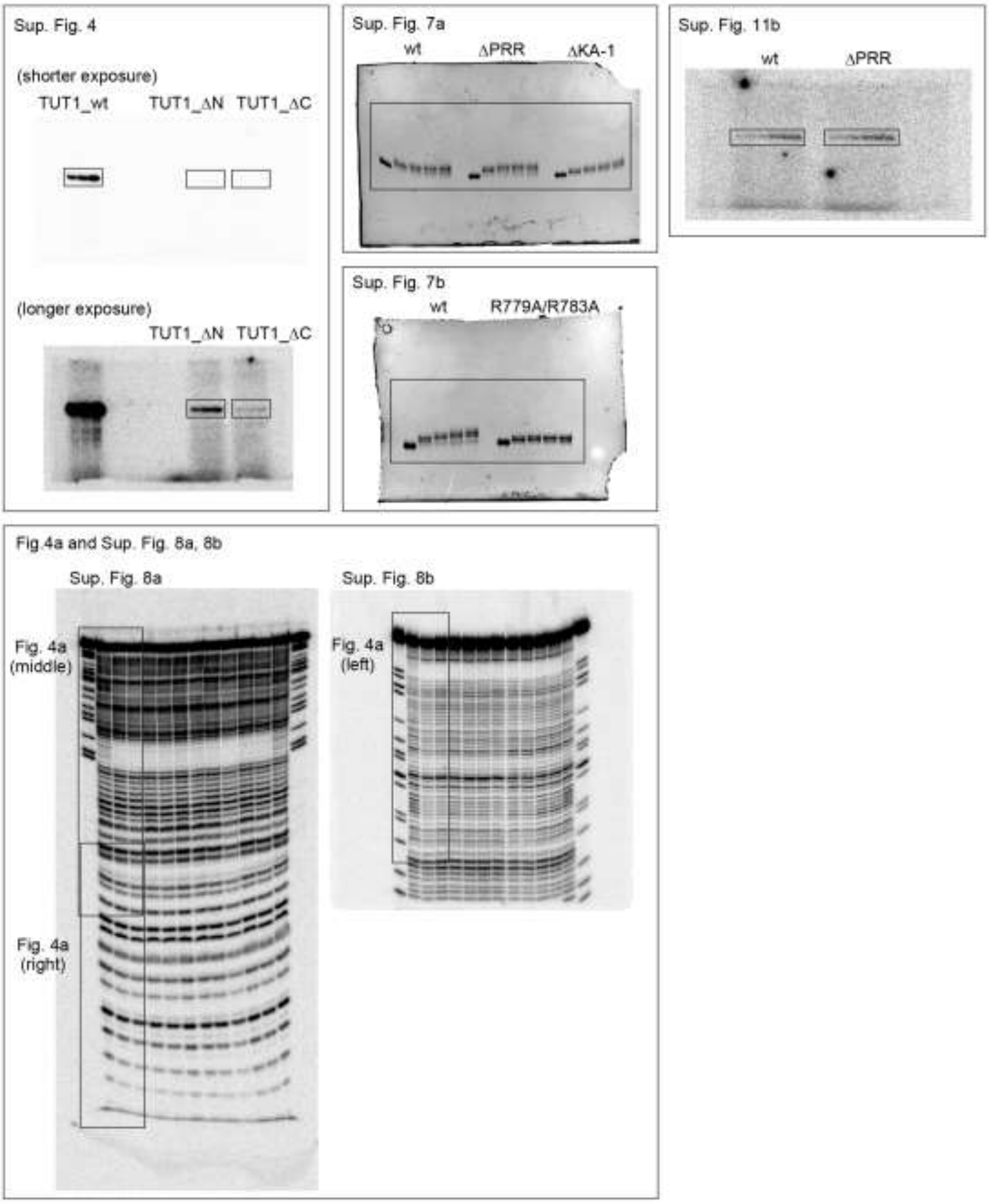

Supplementary Fig. 14: Uncropped images of Fig. 4, Supplementary Figs. 4, 7, 8, and 11b.

Supplementary Table 1: nucleotide sequence of synthetic TUT1 gene

TUT1_gene<br>(codon-optimized for<br>*E.coli*)	5'-ATGGCGGCGGTTGATAGCGATGTTGAAAGCCTGCCGCGCGGTGGCTTTCG<br>CTGCTGCCTGTGCCATGTGACCACCGCGAATCGCCCGAGCCTGGATGCACAT<br>CTGGGTGGCCGCAAACATCGTCATCTGGTGGAACTGCGTGCGGCGCGTAAA<br>GCGCAGGGCCTGCGTAGCGTGTTTGTGAGCGGCTTTCCGCGCGATGTGGAT<br>AGCGCGCAGCTGAGCGAATATTTTCTGGCGTTTGGCCCGGTGGCGAGCGTG<br>GTGATGGATAAAGATAAAGGCGTGTTTGCGATTGTGGAAATGGGCGATGTGG<br>GCGCGCGTGAAGCGGTTCTGAGCCAGAGCCAGCATAGCCTGGGCGGTCATC<br>GTCTGCGTGTGCGTCCGCGCGAACAAAAAGAATTTCAGAGCCCGGCAAGC<br>AAAAGCCCGAAAGGCGCAGCGCCGGATAGTCATCAGCTGGCGAAAGCGCT<br>GGCGGAAGCAGCGGATGTGGGTGCGCAGATGATTAAACTGGTGGGCCTGCG<br>TGAACTGAGCGAAGCGGAACGCCAACTGCGCAGCCTGGTGGTGGCGCTGA<br>TGCAGGAAGTGTTTACCGAATTTTTCCGGGCTGCGTGGTGCATCCGTTTGG<br>CAGCAGCATTAACAGCTTTGATGTGCATGGCTGCGATCTGGATCTGTTTCTG<br>GATCTGGGCGATCTGGAAGAACCGCAGCCGGTTCCGAAAGCACCGGAAAG<br>CCCGAGCCTGGATTCTGCACTGGCAAGCCCGCTGGATCCGCAAGCGCTGGC<br>ATGTACCCCGGCAAGTCCGCCGGATAGCCAACCGCCGGCATCTCCGCAAGA<br>TTCTGAAGCACTGGATTTTGAAACCCCGAGCAGCAGCCTGGCACCGCAGAC<br>CCCGGATAGTGCACTGGCAAGTGAAACCCTGGCATCTCCGCAAAGCCTGCC<br>GCCGGCAAGTCCGCTGCTGGAAGATCGTGAAGAAGGCGATCTGGGCAAAG<br>CGAGCGAACTGGCGGAAACCCCGAAAGAAGAAAAAGCGGAAGGCGCGGC<br>GATGCTGGAACTGGTGGGCAGCATTCTGCGTGGCTGTGTGCCGGGCGTGTA<br>TCGTGTTCAGACCGTGCCGAGCGCGCGTCGCCCGGTTGTGAAATTTTGTCAT<br>CGCCCGAGCGGCCTGCATGGCGATGTGAGCCTGAGCAACCGCCTGGCGCTG<br>CATAACAGCCGCTTTCTGAGCCTGTGCAGCGAACTGGATGGTCGCGTTCGC<br>CCGCTGGTTTATACCCTGCGTTGTTGGGCACAGGGTCGTGGTCTGAGCGGTA<br>GCGGTCCGCTGCTGAGTAATTATGCGCTGACCCTGCTGGTTATTTATTTTCTG<br>CAGACCCGTGATCCGCCGGTTCTGCCGACCGTTAGCCAGCTGACCCAGAAA<br>GCGGGCGAAGGTGAACAGGTGGAAGTGGATGGCTGGGATTGCAGCTTCCC<br>GCGCGACGCCAGCCGCCTGGAACCGAGCATTAACGTGGAACCGCTGAGCA<br>GCCTGCTGGCGCAGTTTTTTAGCTGCGTGAGCTGCTGGGATCTGCGTGGCA<br>GCCTGCTGAGCCTGCGTGAAGGTCAAGCGCTGCCGGTTGCGGGTGGTCTGC<br>CGAGCAATCTGTGGGAAGGTCTGCGTCTGGGTCCGCTGAATCTGCAGGATC<br>CGTTTGATCTGAGCCATAACGTGGCGGCGAATGTTACCAGCCGTGTTGCGGG<br>TCGCCTGCAGAATTGCTGCCGTGCGGCGGCGAATTATTGTCGTAGCCTGCAG<br>TATCAGCGTCGTAGCAGCCGTGGTCGTGATTGGGGTCTGCTGCCGCTGCTGC<br>AACCGAGTAGCCCGAGTAGCCTGCTGTCTGCAACCCCGATTCCGCTGCCGC<br>TGGCACCGTTTACCCAACTGACCGCAGCGCTGGTTCAGGTGTTTCGCGAAG<br>CGCTGGGCTGCCATATTGAACAGGCGACCAAACGTACCCGTAGCGAAGGCG<br>GTGGTACCGGCGAAAGCAGCCAGGGTGGCACCAGCAAACGCCTGAAAGTG<br>GATGGCCAGAAAAACTGCTGCGAAGAAGGCAAAGAAGAACAGCAGGGCT<br>GCGCGGGTGATGGCGGCGAAGATCGTGTGGAAGAAATGGTGATTGAAGTGG<br>GCGAAATGGTGCAGGATTGGGCGATGCAGAGCCCGGGTCAACCGGGTGATC<br>TGCCGCTGACCACCGGTAAACATGGCGCACCGGGTGAAGAAGGTCAGCCG<br>AGTCATGCGGCGCTGGCAGAACGTGGCCCGAAAGGTCATGAAGCAGCGCA<br>GGAATGGAGCCAGGGTGAAGCGGGTAAAGGTGCAAGCCTGCCGAGCTCTG<br>CGAGCTGGCGTTGTGCGCTGTGGCATCGTGTTTGGCAGGGTCGTCGTCGTG<br>CGCGTCGTCGTCTGCAGCAGCAAACCAAAGAAGGCGCAGGTGGTGGTGCA<br>GGTACCCGTGCAGGTTGGCTGGCAACCGAAGCACAAGTTACCCAGGAACTG<br>AAAGGCCTGAGCGGCGGTGAAGAACGTCCGGAAACCGAACCGCTGCTGAG<br>CTTTGTGGCGAGCGTGAGCCCGGCGGATCGTATGCTGACCGTGACCCCGCT<br>GCAAGATCCGCAGGGTCTGTTTCCGGATCTGCATCATTTTCTGCAGGTGTTT<br>CTGCCGCAGGCGATTCGCCATCTGAAATAA-3'

Supplementary Table 2: List of synthetic nucleotides

TUT1_Nter_NdeI_Fw	5'-TTTTTTTTCATATGGCGGCGGTTGATAGCGATG-3'
TUT1_FromG54_NdeI_Fw	5'-TTTTTTTTCATATGGGCCTGCGTAGCGTGTTTGTGAG-3'
TUT1_FromG141_NdeI_Fw	5'-TTTTTTTTCATATGGGCGCAGCGCCGGATAGTCATC-3'
TUT1_FromS598_NdeI_Fw	5'-TTTTTTTTCATATGAGTAGCCCGAGTAGCCTGCTG-3'
TUT1_Cter_XhoI_Rv_pET15	5'-TTTTCTCGAGTTATTTCAGATGGCGAATCGCCTG-3'
TUT1_Cter_XhoI_Rv_pET22	5'-TTTTCTCGAGTTTCAGATGGCGAATCGCCTG-3'
TUT1_ToS599_XhoI_Rv	5'-TTTTCTCGAGGCTACTCGGTTGCAGCAGCGGCAGC-3'
TUT1_ToD702_XhoI_Rv	5'-TTTTCTCGAGTTAATCCTGCACCATTTCGCCCAC-3'
TUT1_ToE748_XhoI_Rv	5'-TTTTCTCGAGTTATTCCTGCGCTGCTTCATGACC-3'
TUT1_ToK234_Rv	5'-TTTCGGAACCGGCTGCGGTTCTTCC-3'
TUT1_FromL305_Fw	5'-CTGCCGCCGGCAAGTCCGCTG-3'
TUT1_ToG650_Rv	5'-GCCGGTACCACCGCCTTCGCTACG-3'
TUT1_FromQ751_Fw	5'-CAGGGTGAAGCGGGTAAAGGTGC-3'
TUT1_R779AR783A_Fw	5'-GCAGGGTCGTGCGCGTGCGCGTGCGCGTCTGCAGCAGC-3'
TUT1_R779AR783A_Rv	5'-GCTGCTGCAGACGCGCACGCGCACGCGCACGACCCTGC-3'
TUT1_R871AK874A_XhoI_Rv	5'-TTTTCTCGAGCGCCAGATGCGCAATCGCCTGCGGCAG-3'
TUT1_C372A_Fw	5'-CCGGTTGTGAAATTTGCGCATCGCCCGAGCGGCCT-3'
TUT1_C372A_Rv	5'-AGGCCGCTCGGGCGATGCGCAAATTTCACAACCGG-3'
TUT1_C399A_Fw	5'-CGCTTTCTGAGCCTGGCGAGCGAACTGGATGGTCG-3'
TUT1_C399A_Rv	5'-CGACCATCCAGTTCGCTCGCCAGGCTCAGAAAGCG-3'
TUT1_C415A_Fw	5'-GGTTTATACCCTGCGTGCGTGGGCACAGGGTCGTG-3'
TUT1_C415A_Rv	5'-CACGACCCTGTGCCCACGCACGCAGGGTATAAACC-3'
TUT1_C501AC504S_Fw	5'-CAGTTTTTTAGCGCGGTGAGCAGCTGGGATCTGCG-3'
TUT1_C501AC504S_Rv	5'-CGCAGATCCCAGCTGCTCACCGCGCTAAAAAACTG-3'
TUT1_C574A_Fw	5'-TGCGGCGGCGAATTATGCGCGTAGCCTGCAGTATC-3'
TUT1_C574A_Rv	5'-GATACTGCAGGCTACGCGCATAATTCGCCGCCGCA-3'
TUT1_Seq_500	5'-GTGCGCAGATGATTAAACTGG-3'
TUT1_Seq_1000	5'-GCGGAAGGCGCGGCGATGCTG-3'
TUT1_Seq_1500	5'-GCTGGCGCAGTTTTTTAGCTG-3'
TUT1_Seq_2000	5'-GTGGATGGCCAGAAAAACTGC-3'

SUPPLEMENTARY REFERENCES

1. Sickmier, E.A. et al. Structural basis for polypyrimidine tract recognition by the essential pre-mRNA splicing factor U2AF65. *Mol Cell* **23**, 49-59 (2006).
2. Lunde, B.M., Magler, I. & Meinhart, A. Crystal structures of the Cid1 poly (U) polymerase reveal the mechanism for UTP selectivity. *Nucleic Acids Res* **40**, 9815-9824 (2012).
3. Yates, L.A. et al. Structural basis for the activity of a cytoplasmic RNA terminal uridylyl transferase. *Nat Struct Mol Biol* **19**, 782-7 (2012).
4. Munoz-Tello, P., Gabus, C. & Thore, S. Functional implications from the Cid1 poly(U) polymerase crystal structure. *Structure* **20**, 977-86 (2012).
5. Munoz-Tello, P., Gabus, C. & Thore, S. A critical switch in the enzymatic properties of the Cid1 protein deciphered from its product-bound crystal structure. *Nucleic Acids Res* **42**, 3372-80 (2014).
